# UK Medical Cannabis Registry: an updated analysis of clinical outcomes of medicinal cannabis therapy for hypermobility-associated chronic pain

**DOI:** 10.1007/s10067-026-08166-z

**Published:** 2026-05-30

**Authors:** Mariam Alemi, Simon Erridge, Evonne Clarke, Katy McLachlan, Ross Coomber, Shelley Barnes, Alia Darweish Medniuk, Rahul Guru, Wendy Holden, Mohammed Sajad, Robert Searle, Azfer Usmani, Sanjay Varma, James J. Rucker, Michael Platt, Mikael H. Sodergren

**Affiliations:** 1 Medical Cannabis Research Group, Imperial College London, London, UK; 2 Curaleaf Clinic, London, UK; 3 St George’s University Hospitals NHS Foundation Trust , London, UK; 4 Department of Psychological Medicine, King’s College London, London, UK; 5 South London & Maudsley NHS Foundation Trust, London, UK; 6 Guy’s & St Thomas’ NHS Foundation Trust, London, UK; 7 Department of Surgery & Cancer, Imperial College London, 1st Floor, Block B, Hammersmith Hospital, Du Cane Road, London, W12 0HS UK

**Keywords:** Chronic pain, Ehlers-Danlos syndrome, Hypermobility spectrum disorders, Medical cannabis, Patient-reported outcomes

## Abstract

**Introduction/Objective:**

The primary aim of this study was to evaluate changes in pain-specific and general health-related quality of life in individuals prescribed cannabis-based medicinal products (CBMPs) for hypermobility-associated chronic pain.

**Methods:**

The case series utilised data from the UK Medical Cannabis Registry. Primary outcomes were changes in Brief Pain Inventory (BPI), Pain Visual Analogue Scale (VAS), Short-Form McGill Pain Questionnaire-2 (SF-MPQ-2), EQ-5D-5L index value, Generalised Anxiety Disorder-7 (GAD-7), and Single-item Sleep Quality Scale (SQS) over 24 months. Repeated measures analysis of variance was used to assess changes over time, with post hoc pairwise comparisons performed for significant findings.

**Results:**

A total of 240 patients were analysed. Changes were observed across all patient-reported outcome measures (PROMs) on repeated measures analysis of variance (*p* < 0.001). Post hoc pairwise comparisons for the BPI subscales, SF-MPQ-2 and Pain VAS demonstrated improvement from baseline to all subsequent timepoints (*p* < 0.001). By 24 months, 56.67% (*n* = 136) and 61.25% (*n* = 147) of participants reported clinically significant improvements in BPI severity and interference respectively. Clinically significant improvements were also reported for SF-MPQ-2 (47.08%, *n* = 113) and Pain VAS scores (60.00%, *n* = 144).

**Conclusion:**

In this real-world cohort, CBMP treatment was associated with sustained improvements in outcomes for individuals with hypermobility-associated chronic pain. These findings support the need for further controlled studies to determine causality.

**Table:** 

**Key Points** • *This 24-month real-world study demonstrates sustained improvements in pain, anxiety, and sleep outcomes for patients with hypermobility-associated chronic pain treated with cannabis-based medicinal products, with approximately 60% achieving clinically meaningful pain reductions*. • *Cannabis-based medicinal products were associated with reductions in concomitant opioid prescriptions at 12, 18, and 24 months*. • *This represents the largest and longest-duration observational study of medical cannabis therapy specifically in hypermobility spectrum disorders and Ehlers-Danlos syndrome, addressing a critical evidence gap in chronic pain management*. • *Adverse events were predominantly mild-to-moderate in severity, with poor baseline sleep quality and current cannabis use identified as positive predictors of pain improvement, informing patient selection and treatment optimisation*.

**Supplementary Information:**

The online version contains supplementary material available at 10.1007/s10067-026-08166-z.

## Introduction

Hypermobility spectrum disorders (HSD) and Ehlers-Danlos syndrome (EDS) are heritable connective tissue disorders characterised by joint hypermobility and complications including dislocation and subluxation, vascular fragility, and multi-system connective tissue abnormalities [1]. Systemic manifestations include disabling chronic pain, gastrointestinal dysfunction, and dysautonomia [1].

Chronic pain affects nearly all patients with hypermobile EDS (hEDS) or HSD. Up to 90% report generalised pain, 80% experience joint pain, and 68% have symptoms consistent with neuropathic pain [2]. Pain arises from a combination of nociceptive, neuropathic, and nociplastic mechanisms [3].

Consequently, effective pharmaceutical options for hypermobility-associated chronic pain are scarce. Only 40% of individuals are reported to achieve improvements in pain severity with non-steroidal anti-inflammatory (NSAID) or opioid use, and just 25% from paracetamol [4]. Associated risks of using these agents in chronic pain include gastrointestinal side effects, orthostatic hypotension, tolerance, paradoxical hyperalgesia and risk of opioid use disorder (OUD) [4]. Similarly, neuropathic agents have variable efficacy, particularly given the poorly defined pain pathophysiology in hEDS/HSD [5].

The endocannabinoid system (ECS) modulates pain signalling through regulation of excitatory/inhibitory networks and synaptic plasticity, particularly relevant in chronic pain where central sensitisation leads to dysregulated pain processing [6]. It comprises primarily of G-protein-coupled cannabinoid type 1 (CB1) and type 2 (CB2) receptors, and their endogenous ligands, anandamide (AEA) and 2-arachidonoylglycerol (2-AG) [6]. These endocannabinoids act as retrograde neurotransmitters, modulating presynaptic release of excitatory neurotransmitters such as glutamate [7].

Phytocannabinoids from *Cannabis sativa,* primarily (−)-trans-Δ^9^-tetrahydrocannabinol (THC) and cannabidiol (CBD), interact with the ECS and extended ECS to modulate pain and inflammation [6]. THC is a partial agonist at the CB1 and CB2 receptors, influencing nociceptive transmission within spinal pathways and supraspinal circuits, involved in emotional and cognitive pain processing [8]. CB2 activation on immune and glial cells exert antinociceptive and anti-inflammatory effects and may also enhance peripheral opioid signalling [8]. CBD has relatively low orthosteric affinity for these receptors but enhances endocannabinoid tone by inhibiting AEA degradation via fatty acid binding proteins (FABPs) and fatty acid amide hydrolase (FAAH) [9]. It also influences nociceptive processing through mediation of serotonin 5-hydroxytryptamine 1 A and transient receptor potential cation channel subfamily V member 1 (TRPV1) channels, contributing also to CBD’s anxiolytic properties [9].

Preclinical models consistently demonstrate CB1/CB2-mediated antinociceptive and anti-inflammatory effects in animal models, providing mechanistic rationale for the investigation of CBMPs in chronic pain [10].

A 2021 review of 32 randomised controlled trials (RCTs) in chronic non-cancer pain populations found a 7% increase in patients achieving ≥ 30% pain improvement with CBMPs compared to placebo (risk difference 0.07, 95% CI: 0.02–0.12), alongside clinically meaningful improvements in physical functioning and sleep quality [11]. However, no patients with HSD/hEDS were included, revealing a key gap in the evidence-base [11]. A meta-analysis by Barakaji et al*.* similarly supported CBMPs’ analgesic potential, reporting greater improvements in pain severity versus placebo [12], although effect sizes were modest—likely reflecting heterogenous dosing regimens and CBMP formulations. RCTs investigating nabiximols, an oromucosal spray containing 2.7 mg THC and 2.5 mg CBD per spray, showed that approximately 1 in 2 patients achieved the ≥ 30% pain reduction threshold [13]. Importantly, many of these studies were underpowered, short in duration, or focussed on acute pain conditions, limiting their applicability to the chronic pain phenotype in HSD/hEDS [13].

Data from previous UKMCR studies highlight improvements in pain and quality of life with CBMP treatment in HSD/hEDS patients [14], albeit over shorter durations and with smaller samples sizes than the present study. Long-term, hypermobility-specific evidence for CBMP efficacy and safety remains lacking within CBMP literature.

This study aims to address this gap by assessing changes in pain-specific and general patient-reported outcome measures (PROMs) in individuals with HSD/hEDS prescribed CBMPs as the primary outcome, and by evaluating prevalence and severity of CBMP-related adverse events over 24-months as the secondary outcome.

## Methods

### Study design

This case series spans 24 months, using data extracted from the UK Medical Cannabis Registry (UKMCR) from patients with HSD/hEDS. Questionnaires were completed at baseline and 1, 3, 6, 12, 18 and 24 months post-enrolment by consenting patients, to assess health-related outcomes over time.

### Setting and participants

All study participants are registered in the UKMCR, which is a patient registry collecting data from patients who are prescribed CBMPs in the UK and Crown Dependencies. Patients were enrolled consecutively, and the UKMCR has received overarching ethical approval by the Central Bristol Research Ethics Committee (reference: 22/SW/0145). Participants were over 18 years old with a primary diagnosis of HSD/hEDS for which they are prescribed CBMPs. They were all required to have been enrolled at least 2 years prior to when the data was extracted, on 6th January 2025. Exclusion criteria included incomplete baseline PROM assessment and hypermobility being a secondary or tertiary indication for treatment with CBMPs. All CBMPs were manufactured in accordance with Good Manufacturing Practice (GMP) standards.

### Data collection

Demographic data was recorded from baseline assessment by clinicians, including age, sex, occupation and body mass index (BMI) calculated from patient height and weight. Prevalence of relevant comorbid conditions and age was used to calculate the Charlson Comorbidity Index [15]. Tobacco and alcohol data were recorded including smoking status, pack-years, and weekly alcohol consumption in units. Cannabis status prior to starting treatment with CBMPs was obtained. For those who had previous or current cannabis use, lifetime cannabis consumption was determined using gram-years. The route of administration and current consumption in grams was quantified for current consumers.

Patient prescriptions were also recorded, including regular medications and CBMP prescriptions (formulations, route of administration, and CBD/THC strains and concentrations). Oral morphine equivalents (OME) (mg/day) of prescribed opioids and pregabalin equivalents (PGE) (mg/day) of prescribed gabapentinoids were calculated to look at changes in medication use at each follow-up interval, using conversion values provided by the British National Formulary and the Faculty of Pain Medicine [16]. Use of non-opioid analgesics available without prescription and engagement with non-pharmacological interventions such as physical therapy or physiotherapy were not systematically captured by the UKMCR and are therefore not included in the present analysis.

### Patient reported outcome measures (PROMs)

#### Brief Pain Inventory (BPI) short form

The BPI provides quantitative assessment of both pain intensity and pain interference using numerical rating scales (NRS) from 0–10 [17]. A 1-point decrease in BPI is considered the minimal clinically important difference (MCID) for both pain subscales [18].

#### Short form—McGill Pain Questionnaire 2 (SF-MPQ-2)

The SF-MPQ-2 is a questionnaire consisting of 22 pain descriptors across 4 categories: continuous pain, intermittent pain, neuropathic pain and affective pain. Each descriptor is scored from ‘0’ = ‘no pain’ to ‘10’ = ‘worst possible pain’ [19]. A mean of these scores can produce an overall pain score. A 1-point decrease is considered the MCID for this scale [18].

#### Pain Visual Analogue Scale (Pain VAS)

Pain VAS is a visual single-item scale from ‘no pain’ (score 0) to ‘worst possible pain’ (score 10), capturing the respondents’ current pain intensity [20]. A 1-point decrease is considered the MCID for this scale [18].

#### EQ-5D-5L

The EQ-5D-5L measures health decrements. The first part of the tool defines health in 5 dimensions: Mobility, Self-care, Usual Activities, Pain/Discomfort, and Anxiety/Depression, where each dimension is rated from 1 to 5 (‘1’ = ‘no problem’ and ‘5’ = ‘extreme problems’) [21]. Completion generates a 5-digit score that corresponds to a country-specific 5L index value, which indicates health-related quality of life [22].

#### Generalised Anxiety Disorder 7 (GAD-7)

GAD-7 is a 7-item anxiety scale facilitating assessment of recent anxiety symptoms and their severity from ‘0’ = ‘not at all’ to ‘3’ = ‘nearly every day’. Total score out of 21 determines degree of anxiety: 5–9 mild, 10–14 moderate, and ≥ 15 severe anxiety [23]. A 4-point decrease is considered the MCID for GAD-7 [24].

#### Single-item Sleep Quality Scale (SQS)

The SQS captures patient-reported sleep quality over the last 7 days, utilising a single scale from ‘0’ = ‘terrible’ sleep quality to ‘10’ = ‘excellent’ sleep quality [25]. A 2.6-point increase is considered the MCID for SQS [25].

#### Patient Global Impression of Change (PGIC)

The PGIC assesses patient impressions of health changes over the course of a treatment from 1 to 7, where ‘1’ = ‘no change or worse’, and ‘7’ = ‘a great deal better’ [26].

### Adverse events

Adverse events were also recorded either by patients contemporaneously or when prompted during completion of PROMs. Clinicians could also report adverse events during appointments if otherwise still unreported. These were classified using the Common Terminology Criteria for Adverse Events v4.0 [27].

### Primary and secondary outcomes

Primary outcomes of this study were changes across the studied period in the following PROMs: BPI, SF-MPQ-2, Pain VAS, EQ-5D-5L index value, GAD-7, and SQS, as well as the prevalence and severity of adverse events. Secondary outcomes included changes in concomitantly prescribed opioids and gabapentinoids.

### Missing data

Missing PROM data at follow-ups were addressed using multiple imputation by chained equations (MICE) in R.

### Statistical analysis

Descriptive statistics were used to summarise the data whereby parametric data are presented as mean (± standard deviation; SD) and non-parametric data are presented as median (interquartile range; IQR). Frequencies were reported as n (percentage). PROM data were analysed at intervals of baselines, 1, 3, 6, 12, 18 and 24 months. These were assessed using a repeated measures analysis of variance (ANOVA). If the repeated measures ANOVA was statistically significant, a post-hoc pairwise comparison of time-periods for each PROM was planned with Bonferroni correction.

Both univariable and multivariable logistic regression models were used to identify clinical variables associated with positive changes in PROM score, where clinically significant change was defined by MCID threshold, or a positive improvement if an MCID does not exist. Independent variables in the multivariable logistic regression models included age, sex, body mass index, prior cannabis exposure, CBMP formulation (oil, dried flower, combination, other), THC and CBD dose modelled as quartiles, and baseline PROM scores. Results from the models were presented as odds ratios with corresponding p-values, identifying predictors of PROM improvement at varying degrees of statistical significance. Statistical significance for all analyses was set at a p-value of < 0.050. All analyses were performed using R (v4.5.0; R Core Team, Vienna, Austria) within RStudio (v2024.12.1 + 563; Posit Software, MA, USA).

## Results

### Eligibility

**Fig. 1:**
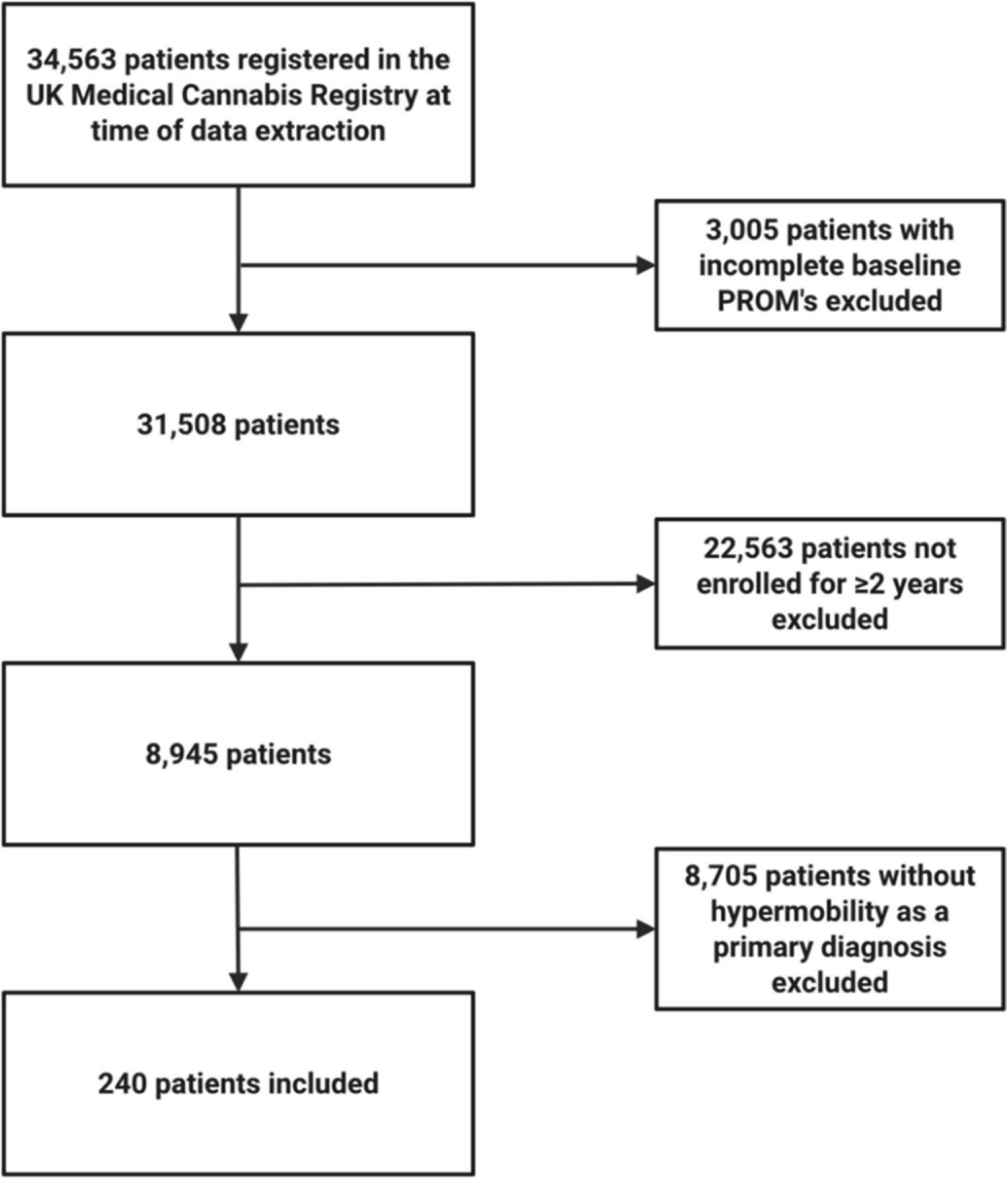
Patient eligibility flow chart showing the inclusion and exclusion of patients from the UK Medical Cannabis Registry

A total of 34,563 patients registered in the UKMCR were assessed for eligibility. Excluding patients with incomplete baseline PROMs, insufficient enrolment duration (< 2 years), or if hypermobility was not their recorded primary diagnosis, 240 of 34,563 patients (0.69%) were eligible for inclusion (Fig. 1).

### Baseline demographics

Baseline demographics of participants are shown in Table 1. Mean participant age was 37.83 (± 10.59) years, and females accounted for 80.83% (*n* = 194) of the cohort. Occupation statistics showed the greatest number of participants were ‘unemployed’ (*n* = 118; 49.17%). The mean participant BMI was 27.00 (± 7.01) kg/m^2^. The median Charlson Comorbidity Index was 1.00 (IQR 1.00–2.00). 49.17% (*n* = 118) of participants had never smoked tobacco, 35.00% (*n* = 84) were ex-smokers (8.31 ± 7.70 pack years), and 15.83% were current smokers (12.17 ± 16.30 pack years). Regarding cannabis use, 49.17% (*n* = 118) were current cannabis users and 14.17% (*n* = 34) were previous users, whilst 36.67% (*n* = 88) were cannabis naïve prior to starting treatment with CBMPs.

**Table 1:** Patient demographics and baseline characteristics (*n* = 240)

Characteristic	n (%) or Mean ± SD
Age	37.83 ± 10.59
Sex	
Male	46 (19.17)
Female	194 (80.83)
BMI (kg/m^2^)	27.00 ± 7.01
Weekly Alcohol Units	2.45 ± 8.35
Tobacco status	
Never smoked	118 (49.17)
Ex-smoker	84 (35.00)
Current smoker	38 (15.83)
Cannabis status	
Never used	88 (36.67)
Ex-user	34 (14.17)
Current user	118 (49.17)
Occupation	
Armed forces occupations	1 (0.42)
Clerical support workers	8 (3.33)
Craft and related trades workers	4 (1.67)
Elementary occupations	2 (0.83)
Managers	9 (3.75)
NR	8 (3.33)
Other occupations	44 (18.33)
Professional	29 (12.08)
Service and sales workers	10 (4.17)
Skilled agricultural, forestry and fishery workers	1 (0.42)
Technicians and associate professionals	6 (2.50)
Unemployed	118 (49.17)

*BMI* body mass index, *NR* not recorded, *SD* standard deviation

### CBMP formulations and doses

At baseline, CBMPs were prescribed as medium-chain triglyceride oils (n = 221; 92.08%) and dried flower (*n* = 123; 51.25%). By 24 months the proportion of patients using oils had declined to 75.00% (*n* = 180; Supplementary Fig. 1). At baseline, median CBD dose was 20.00 (IQR 2.00–50.05) mg/day, and median THC dose was 7.50 (IQR 1.00–21.00) mg/day. At 24 months these were 26.05 (IQR 16.50–66.08) mg/day and 125.50 (IQR 19.31–244.50) mg/day respectively, highlighting dosing adjustments to account for tolerance and individualised symptoms (Supplementary Fig. 2).

### PROMs

**Fig. 2:**
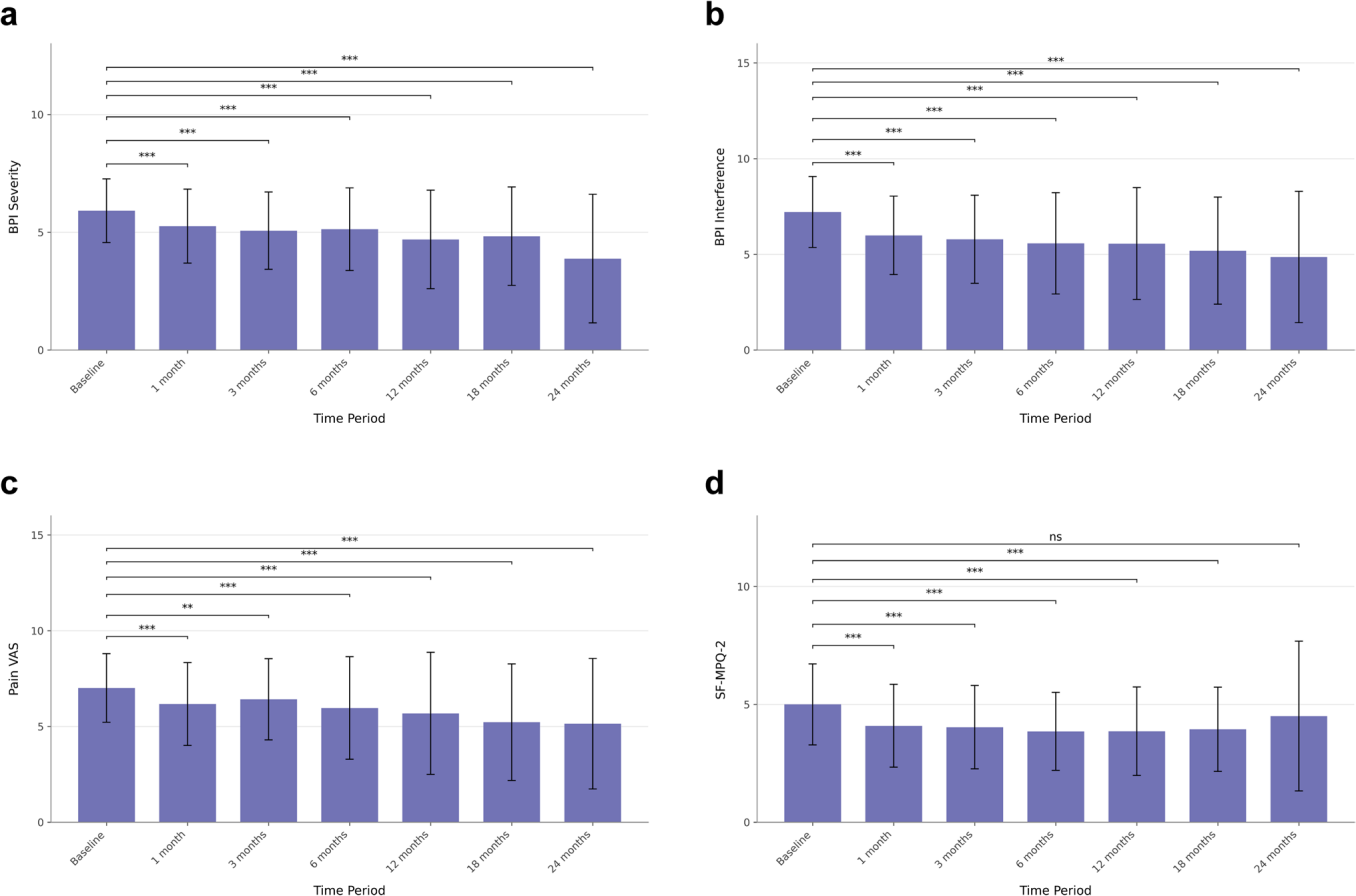
Longitudinal trajectories of patient-reported outcome measures over 24 months. Mean (± SD) values for the a) Brief Pain Inventory (BPI) Severity and b) Interference subscales, c) Pain Visual Analogue Scale (Pain VAS), and d) Short-Form McGill Pain Questionnaire-2 (SF-MPQ-2), are plotted at baseline, 1, 3, 6, 12, 18, and 24 months. *p < 0.050; **p < 0.010; ***p < 0.001; ns – non-significant

Repeated-measures ANOVA demonstrated changes over time for all pain-related PROMs: BPI severity and interference, SF-MPQ-2 and Pain VAS scores (*p* < 0.001; Table 2). Post-hoc pairwise comparisons for these PROMs showed improvement from baseline at most follow-up intervals (*p* < 0.001; Table 3; Fig. 2). BPI Interference showed the greatest reduction at 24 months (mean difference: −2.36 ± 3.91; *p* < 0.001), with Pain VAS (mean difference: −1.87 ± 3.73; *p* < 0.001) and BPI Severity (mean difference: −2.03 ± 2.93; *p* < 0.001) also showing sustained improvements to 24 months. SF-MPQ-2 scores consistently improved up to 18 months (*p*<0.001), but did not differ from baseline at 24 months (mean difference: −0.50 ± 3.60; *p* = 0.682). EQ-5D-5L index values improved at every follow-up point compared with baseline (*p* < 0.001), whilst SQS scores also improved, up to 24 months, *p* < 0.010. GAD-7 scores reduced up to 6 months (*p* < 0.001).

**Table 2:** Mean ± standard deviation (SD) for pain-related patient reported outcome measures (PROMs) at each follow-up interval

PROM	Baseline	1 month	3 months	6 months	12 months	18 months	24 months	*p*-value
BPI Interference	7.21 ± 1.85	5.99 ± 2.05	5.78 ± 2.30	5.57 ± 2.65	5.56 ± 2.92	5.19 ± 2.80	4.86 ± 3.43	< 0.001
BPI Severity	5.91 ± 1.35	5.26 ± 1.57	5.07 ± 1.64	5.13 ± 1.75	4.69 ± 2.09	4.83 ± 2.09	3.88 ± 2.73	< 0.001
Pain VAS	7.01 ± 1.79	6.17 ± 2.16	6.42 ± 2.12	5.96 ± 2.68	5.68 ± 3.19	5.22 ± 3.05	5.14 ± 3.41	< 0.001
SF-MPQ-2	5.00 ± 1.72	4.09 ± 1.76	4.03 ± 1.77	3.85 ± 1.66	3.86 ± 1.88	3.94 ± 1.79	4.50 ± 3.18	< 0.001
EQ-5D-5L Index value	0.22 ± 0.29	0.42 ± 0.26	0.47 ± 0.27	0.44 ± 0.29	0.41 ± 0.32	0.42 ± 0.33	0.42 ± 0.38	< 0.001
GAD-7	9.06 ± 6.10	6.66 ± 5.10	6.70 ± 5.32	7.67 ± 6.48	8.27 ± 6.84	7.41 ± 6.10	7.69 ± 7.02	< 0.001
SQS	3.67 ± 2.24	5.31 ± 2.58	5.50 ± 2.64	4.96 ± 2.87	5.36 ± 3.13	5.76 ± 3.16	4.68 ± 3.45	< 0.001

*BPI* Brief Pain Inventory Short Form, *Pain VAS* Pain Visual Analogue Scale, *SF-MPQ-2* Short form-McGill Pain Questionnaire 2, *GAD-7* Generalised Anxiety Disorder-7, *SQS* Single-item Sleep Quality Scale. Patients with at least one completed PROM at each interval, n (%): Baseline 240 (100.00); 1 month 210 (87.50%); 3 months 186 (77.50%); 6 months 158 (65.83%); 12 months 135 (56.25%); 18 months 111 (46.25%); 24 months 90 (37.50%). Missingness handled via multiple imputation.

**Table 3:** Summary of pairwise comparisons between baseline and follow-up points for patient-reported outcome measures (PROMs)

PROM	Months	Mean Difference ± SD	*p*-value	Significance
BPI Interference	1 month	1.22 ± 1.94	< 0.001	***
	3 months	1.43 ± 2.11	< 0.001	***
	6 months	1.64 ± 2.80	< 0.001	***
	12 months	1.65 ± 2.92	< 0.001	***
	18 months	2.03 ± 3.13	< 0.001	***
	24 months	2.36 ± 3.91	< 0.001	***
BPI Severity	1 month	0.65 ± 1.47	< 0.001	***
	3 months	0.84 ± 1.46	< 0.001	***
	6 months	0.78 ± 1.67	< 0.001	***
	12 months	1.22 ± 2.16	< 0.001	***
	18 months	1.01 ± 2.33	< 0.001	***
	24 months	2.03 ± 2.93	< 0.001	***
Pain VAS	1 month	0.84 ± 2.23	< 0.001	***
	3 months	0.59 ± 2.21	0.001	**
	6 months	1.05 ± 2.55	< 0.001	***
	12 months	1.33 ± 3.48	< 0.001	***
	18 months	1.79 ± 3.37	< 0.001	***
	24 months	1.87 ± 3.73	< 0.001	***
SF-MPQ-2	1 month	0.91 ± 1.54	< 0.001	***
	3 months	0.97 ± 1.58	< 0.001	***
	6 months	1.15 ± 1.59	< 0.001	***
	12 months	1.14 ± 1.98	< 0.001	***
	18 months	1.06 ± 2.28	< 0.001	***
	24 months	0.50 ± 3.60	0.682	ns
EQ-5D-5L index value	1 month	−0.20 ± 0.28	< 0.001	***
	3 months	−0.25 ± 0.32	< 0.001	***
	6 months	−0.22 ± 0.32	< 0.001	***
	12 months	−0.19 ± 0.38	< 0.001	***
	18 months	−0.19 ± 0.38	< 0.001	***
	24 months	−0.20 ± 0.45	< 0.001	***
GAD-7	1 month	2.40 ± 5.24	< 0.001	***
	3 months	2.35 ± 6.36	< 0.001	***
	6 months	1.39 ± 7.98	0.159	ns
	12 months	0.79 ± 8.11	1.000	ns
	18 months	1.65 ± 8.36	0.054	ns
	24 months	1.37 ± 9.37	0.519	ns
SQS	1 month	−1.64 ± 2.80	< 0.001	***
	3 months	−1.83 ± 3.20	< 0.001	***
	6 months	−1.29 ± 3.50	< 0.001	***
	12 months	−1.69 ± 3.59	< 0.001	***
	18 months	−2.09 ± 3.88	< 0.001	***
	24 months	−1.01 ± 4.26	0.006	*

Mean Difference ± standard deviation (SD) reported for baseline minus follow up period. *BPI* Brief Pain Inventory Short Form, *Pain VAS* Pain Visual Analogue Scale, *SF-MPQ-2* Short form-McGill Pain Questionnaire 2, *GAD-7* Generalised Anxiety Disorder-7, *SQS* Single-item Sleep Quality Scale.

*p < 0.050; **p < 0.010; ***p < 0.001, *ns* non-significant

MCID thresholds were met at 24 months by 61.25% (*n* = 147) and 56.67% (*n* = 136) of participants in BPI Interference and Severity, and 47.08% (*n* = 113) reaching SF-MPQ-2 thresholds. Pain VAS showed 60% (*n* = 144) of participants reaching the MCID improvement by the end of the study period. The median PGIC values were 5.00 [4.00–6.00], 5.00 [4.00–6.00], 6.00 [5.00–6.00], 6.00 [4.00–7.00], 6.00 [3.00–7.00], and 6.00 [2.00–7.00] at 1, 3, 6, 12, 18, and 24 months respectively.

### Concomitant opioid and gabapentinoid use

Mean prescribed OME was 51.19 (± 107.74) mg/day at baseline. Reductions in opioid doses were observed at 12, 18 and 24 months (*p* < 0.050). However, no changes in PGE doses were seen between baseline and 24 months (*p* = 1).

### Adverse events

In total, 743 adverse events were reported by 25.00% (*n* = 60) of participants, of which headaches (22.08%; n = 53), fatigue (20.00%; *n* = 48) and lethargy (18.33%; *n* = 44) were most common (Table 4 & Supplementary Fig. 3). Adverse events were classified as mild (*n* = 306; 41.18%), moderate (*n* = 317; 42.67%), severe (*n* = 118; 15.88%), or life-threatening (*n* = 2; 0.27%).

**Table 4:** Frequency and percentage of adverse events with severities. Frequency (%) reported as a proportion of total cohort (n=240). Severity (%) reported as a proportion of the total number of patients with each adverse event

Adverse Event	Frequency (%)	Mild (%)	Moderate (%)	Severe (%)	Life-threatening (%)
Headache	53 (22.08)	15 (28.30)	19 (35.85)	19 (35.85)	0 (0.00)
Fatigue	48 (20.00)	8 (16.67)	23 (47.92)	17 (35.42)	0 (0.00)
Lethargy	44 (18.33)	17 (38.64)	27 (61.36)	0 (0.00)	0 (0.00)
Nausea	43 (17.92)	24 (55.81)	17 (39.53)	2 (4.65)	0 (0.00)
Somnolence	38 (15.83)	0 (0.00)	33 (86.84)	5 (13.16)	0 (0.00)
Dizziness	36 (15.00)	12 (33.33)	14 (38.89)	10 (27.78)	0 (0.00)
Dry Mouth	36 (15.00)	33 (91.67)	3 (8.33)	0 (0.00)	0 (0.00)
Dyspepsia	36 (15.00)	22 (61.11)	13 (36.11)	1 (2.78)	0 (0.00)
Insomnia	35 (14.58)	6 (17.14)	17 (48.57)	12 (34.29)	0 (0.00)
Concentration Impairment	32 (13.33)	18 (56.25)	12 (37.50)	2 (6.25)	0 (0.00)
Abdominal Pain	26 (10.83)	11 (42.31)	12 (46.15)	3 (11.54)	0 (0.00)
Vertigo	25 (10.42)	13 (52.00)	7 (28.00)	5 (20.00)	0 (0.00)
Ataxia	21 (8.75)	11 (52.38)	9 (42.86)	1 (4.76)	0 (0.00)
Generalised Muscle Weakness	21 (8.75)	5 (23.81)	6 (28.57)	10 (47.62)	0 (0.00)
Constipation	19 (7.92)	13 (68.42)	6 (31.58)	0 (0.00)	0 (0.00)
Cognitive Disturbance	18 (7.50)	10 (55.56)	8 (44.44)	0 (0.00)	0 (0.00)
Anorexia	17 (7.08)	5 (29.41)	10 (58.82)	2 (11.76)	0 (0.00)
Blurred Vision	17 (7.08)	6 (35.29)	8 (47.06)	3 (17.65)	0 (0.00)
Pharyngitis	15 (6.25)	1 (6.67)	14 (93.33)	0 (0.00)	0 (0.00)
Tremor	14 (5.83)	7 (50.00)	3 (21.43)	4 (28.57)	0 (0.00)
Weight Loss	14 (5.83)	10 (71.43)	4 (28.57)	0 (0.00)	0 (0.00)
Vomiting	13 (5.42)	9 (69.23)	3 (23.08)	1 (7.69)	0 (0.00)
Confusion	12 (5.00)	8 (66.67)	2 (16.67)	2 (16.67)	0 (0.00)
Diarrhoea	12 (5.00)	4 (33.33)	6 (50.00)	2 (16.67)	0 (0.00)
Spasticity	10 (4.17)	2 (20.00)	3 (30.00)	5 (50.00)	0 (0.00)
Fall	9 (3.75)	4 (44.44)	5 (55.56)	0 (0.00)	0 (0.00)
Fever	9 (3.75)	8 (88.89)	1 (11.11)	0 (0.00)	0 (0.00)
Amnesia	7 (2.92)	4 (57.14)	3 (42.86)	0 (0.00)	0 (0.00)
Lung Infection	7 (2.92)	0 (0.00)	7 (100.00)	0 (0.00)	0 (0.00)
Delirium	5 (2.08)	3 (60.00)	2 (40.00)	0 (0.00)	0 (0.00)
Dysgeusia	5 (2.08)	4 (80.00)	1 (20.00)	0 (0.00)	0 (0.00)
Rash	5 (2.08)	4 (80.00)	1 (20.00)	0 (0.00)	0 (0.00)
Urinary Tract Infection	5 (2.08)	0 (0.00)	4 (80.00)	1 (20.00)	0 (0.00)
Palpitations	3 (1.25)	0 (0.00)	3 (100.00)	0 (0.00)	0 (0.00)
Anxiety	2 (0.83)	0 (0.00)	1 (50.00)	1 (50.00)	0 (0.00)
Migraine	2 (0.83)	0 (0.00)	1 (50.00)	1 (50.00)	0 (0.00)
Pain	2 (0.83)	1 (50.00)	0 (0.00)	1 (50.00)	0 (0.00)
Vasovagal Reaction	2 (0.83)	0 (0.00)	0 (0.00)	1 (50.00)	1 (50.00)
Other	25 (10.42)	8 (32.00)	9 (36.00)	7 (28.00)	1 (4.00)

### Logistic regression

Univariable and multivariable logistic regression analyses identified clinically relevant predictors of PROM improvement. Full results of the multivariable analysis can be found in Supplementary Tables 1–8.

#### Brief Pain Inventory short form

**Fig. 3:**
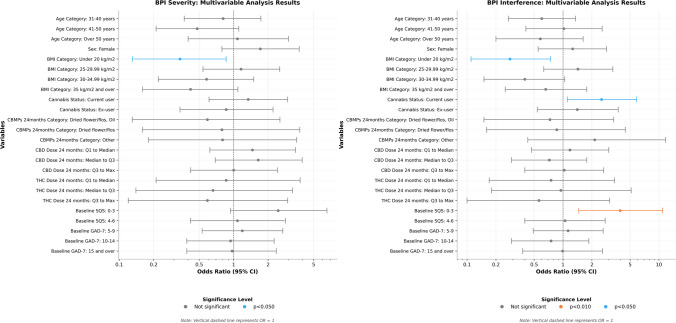
Forest plots showing odds ratios of variables from multivariable logistic regression analysis of Brief Pain Inventory Short Form (BPI) Interference and Severity scores. BMI = Body Mass Index, CBMP = Cannabis-Based Medicinal Product, CBD – Cannabidiol, THC—(−)-trans-Δ^9^-tetrahydrocannabinol, SQS – Single-item Sleep Quality Scale, GAD-7 – Generalised Anxiety Disorder-7, OR – Odds Ratio. *p < 0.050; **p < 0.010; ***p < 0.001; ns – non-significant

Poor baseline sleep quality (SQS 0–3) was associated with better odds of improvement in BPI interference (OR = 3.12, 95% CI: 1.40–7.06, *p* = 0.006) and severity (OR = 2.27, 95% CI: 1.03–5.08, *p* = 0.043), in univariable models. This association was also significant in the multivariable model for BPI Interference (OR = 3.94, 95% CI: 1.46–10.96, *p* = 0.007; Fig. 3), alongside current cannabis use (OR = 2.52, 95% CI: 1.11–5.85, *p* = 0.029). Multivariable analysis of BPI Severity identified that only lower BMI (< 20 kg/m^2^) was associated with lower odds of improvement (OR = 0.34, 95% CI: 0.13–0.86, *p* = 0.025).

#### Short Form—McGill Pain Questionnaire 2

Both univariable and multivariable analyses identified that higher BMI (30–34.99 kg/m^2^) was associated with lower odds of SF-MPQ-2 score improvement (multivariable OR = 0.20, 95% CI: 0.15–0.27, *p* =0.002). Improvement in GAD-7 was predicted by more severe anxiety at baseline in univariable and multivariable models – all score categories (5–9, 10–14, ≥ 15) were associated with higher odds of improvement compared with scores of < 5 (*p* < 0.001).

#### Adverse events

Univariable and multivariable models found that age over 50 years was associated with greater odds of adverse event occurrence (multivariable OR = 3.52, 95% CI: 1.19–10.93, *p* = 0.025).

## Discussion

This study evaluated changes in pain-specific and general health-related quality of life among individuals prescribed CBMPs through the UKMCR for hypermobility-associated chronic pain. Findings contribute real-world evidence for the therapeutic potential of CBMPs in HSD/hEDS, shown by sustained improvements across all PROMs. MCID thresholds were also met by substantial proportions of the cohort at several follow-up intervals, highlighting statistical and clinical significance. A notable reduction in opioid doses at 12, 18, and 24 months suggests an additional role for CBMPs in the development of novel opioid-sparing strategies for pain management, particularly in chronic pain populations at an elevated risk of OUD and polypharmacy.

Pain-related outcomes demonstrated sustained improvements, with approximately 1 in 2 participants exceeding MCID thresholds by the end of the study. The current study showed the greatest reduction in BPI Interference (-2.36 at 24 months), compared with a 0.57-point decrease in the HSD cohort at 18 months [14]. This may reflect differences in study duration, methods of handling missing data and variations in CBMP formulation. Extending treatment duration to 24 months supports the use of CBMPs as a long-term management strategy in chronic pain.

The sustained efficacy of CBMPs over the study period positions them as a favourable analgesic option compared with opioids, where long-term use is constrained by tolerance and risk of OUD [28]. Although dose titration can combat opioid tolerance, this is associated with increasing adverse events [28]. The opioid-sparing potential of CBMPs supports their use as adjuncts to opioid therapy, reducing adverse effects associated with their high doses. THC-mediated mechanisms, such as β-endorphin stimulation and modulation of μ-opioid signalling [29], could explain the observed trend of OME reduction throughout the study. Furthermore, the colocalisation of CB1 and μ-opioid receptors could enhance their analgesic effects. Preclinical studies illustrate THC’s capacity to reduce effective doses of morphine by 3.6 times and codeine by 9.5 times [30]. Beyond analgesia, there is increasing evidence for CBMPs, CBD in particular, as a non-opioid alternative for managing OUD, emphasising the potential of targeting the endocannabinoid system to both mitigate opioid-related harms and provide equally, if not more, efficacious analgesia.

Additionally, CBMPs appear to also have effects on the cognitive and emotional manifestations of pain, in addition to direct effects of transmission of nociceptive signals from the periphery to the cerebral cortex [31]. Individuals with HSD/hEDS are more likely to be affected by anxiety and consequently pain catastrophisation, which has been shown to amplify pain perception. The effects on anxiety and other aspects of health-related quality of life, highlighted in the present study’s results may underlie the reasons why prior studies on musculoskeletal pain suggest that up to 89% of individuals consider CBMPs to be more effective than opioids [32, 33].

The mean THC:CBD ratio of 3.3:1 in this cohort is consistent with high-THC formulations being most frequently associated with analgesic benefit in chronic pain populations [34–36]. The importance of THC:CBD ratios is highlighted in a systematic review by McDonagh et al. [34], which found that only high-THC products were associated with moderate improvements in pain severity and response [34]. This aligns with results of the RCT by Vela et al. [35] which highlighted dose-dependent reductions in pain intensity with THC-containing CBMPs, and with Van de Donk et al. [36] who found the greatest analgesic effects were conferred by high-THC formulations, or products with comparable THC/CBD content. Despite the latter being limited by a small sample size (n = 20), the findings are consistent with those of broader reviews in chronic pain populations. Within the present multivariable analyses, neither CBMP formulation (oil, dried flower, or combination) nor THC and CBD dose quartiles were consistently associated with achievement of MCID thresholds. Several factors may underlie this observation. Doses were individually titrated to symptom control and tolerability, leading to overlapping exposure ranges between responders and non-responders. Nominal prescribed doses do not fully reflect systemic exposure given differences in bioavailability between sublingual oils and inhaled dried flower. Finally, longitudinal changes in formulation, including transition from oils alone to combination regimens, introduce time-varying exposure that is incompletely captured in baseline-stratified models. These findings reinforce the need for prospective studies in which dose and formulation are pre-specified and bioavailability accounted for.

Anxiety outcomes were assessed using GAD-7 scores which improved over the course of 3 months, with over 40% of patients reaching MCID thresholds for anxiety reduction. Evidence relating cannabis use to anxiety is heterogenous, with studies documenting both anxiogenic [37, 38] and anxiolytic effects [39]. For example, a meta-analysis of 31 studies found a positive association between anxiety and cannabis use [37], whilst Myran et al. similarly reported an association between anxiety disorders and cannabis-related hospital visits [38]. Both studies involved unregulated recreational cannabis use, limiting relevance to regulated CBMP prescriptions with clinical oversight.

Focussing on CBMPs, Lichenstein et al. reviewed cannabinoids at different doses and their effects on anxiety, concluding that anxiogenic effects were predominantly seen in uncontrolled high-THC cannabis use, and controlled use of medical THC/CBD formulations was anxiolytic [39]. The authors also acknowledged that CBD’s established anxiolytic properties may attenuate any anxiogenesis induced by THC, likely via serotonergic activity [39]. Additionally, co-administration of THC and CBD synergism may maximise CBMP potential as an analgesic and anxiolytic. These effects are more likely to be observed in CBMPs where THC:CBD ratios are measured and standardised to maximise efficacy, unlike recreational cannabis where THC content is often high and unregulated. However, whilst there may be a pharmacodynamic rationale for CBD protecting against potential neurological and psychiatric side effects associated with CB1 activation by THC, CBD has also been shown to reduce THC metabolism and clearance, resulting in increases in plasma THC concentrations [29]. The complex interplay of pharmacodynamics and pharmacokinetics on an individual’s basis may be a cause of the uncertainty in identifying the optimum CBMP for different pain aetiologies.

The observational, non-randomised design of this study introduces multiple limitations, preventing causal inference. The lack of a control or placebo arm means it is not possible to distinguish treatment effects from placebo-related or natural changes in pain severity. However, as a registry-based study, it provides ecological validity by offering a realistic picture of what CBMP use would look like in routine clinical practice. The study is also susceptible to confounding factors from covariates that were not included in statistical analyses, including concurrent medications or comorbidities.

Although age, body mass index, baseline cannabis exposure, and treatment-related variables were included in multivariable models, additional clinically relevant confounders were not captured by the UKMCR. In particular, engagement with physical therapy and changes in non-opioid pharmacological treatment, such as NSAIDs and paracetamol, were not systematically recorded. Concurrent escalation, discontinuation, or substitution of these treatments may influence PROM trajectories independently of CBMP therapy and represents an important source of unmeasured confounding. Future iterations of the registry will aim to capture these co-interventions to permit more complete adjustment in observational analyses.

The UKMCR records primary indication for treatment but does not systematically capture the criteria used to establish the diagnosis of hEDS or HSD. Consequently, sub-classification between hEDS and HSD is not available to allow complete characterization of the cohort or perform relevant sub-analyses.

There was a substantial proportion of missing data by 24 months, contributing to attrition bias. Multiple imputation was used to handle missing data and performs best when data are missing completely at random. The proportion of participants contributing observed data declined progressively across follow-up, with the greatest attrition occurring between 12 and 24 months. While imputed denominators preserve statistical power, the absolute number of completers should be considered when interpreting the proportions reaching MCID thresholds, as departures from missing-at-random assumptions cannot be fully excluded. Reasons for missing data could include patients not reporting due to symptom resolution, lack of benefit or financial inaccessibility. Nevertheless, multiple imputation is thought to be more resistant to bias than naïve methods of handling missing data or censoring participants [40].

Reliance on PROMs as primary outcomes carries a risk of response and recall biases, particularly given extended periods of time between assessments. The use of self-reported tools provides important patient insight but do not provide objective confirmation of treatment effects.

## Conclusion

This study provides a 24-month real-world evaluation of CBMPs in patients with hypermobility-associated chronic pain. It demonstrates long-term sustained improvement in pain, anxiety and sleep-related outcomes, underpinning health-related quality of life. Despite its observational design, the study provides important insight into potentially addressing an area of significantly unmet therapeutic need. These findings should not be interpreted as advocating for indiscriminate prescription of CBMPs in hypermobility-associated chronic pain. Instead, age > 50 years was associated with greater odds of adverse events and merits closer post-prescription monitoring, particularly for somnolence, fatigue, and orthostatic symptoms. Future randomised controlled trials will be necessary to evaluate the efficacy of CBMPs to determine their future utilisation, without the inherent limitations of observational studies.

## Supplementary Information

Below is the link to the electronic supplementary material.

Supplementary file1 (DOCX 218 KB)

## Data Availability

Data available from the UK Medical Cannabis Registry upon submission and approval of data access request.
