# Supplementary material for: UK Medical Cannabis Registry: an updated analysis of clinical outcomes of medicinal cannabis therapy for hypermobility-associated chronic pain
Source: Clin Rheumatol. 2026 May 30;45(7):4569–82. doi: 10.1007/s10067-026-08166-z (PMC13341818; doi:10.1007/s10067-026-08166-z)
Supplement: Supplementary file 1 — Supplementary file1 (DOCX 218 KB) [file 10067_2026_8166_MOESM1_ESM.docx]

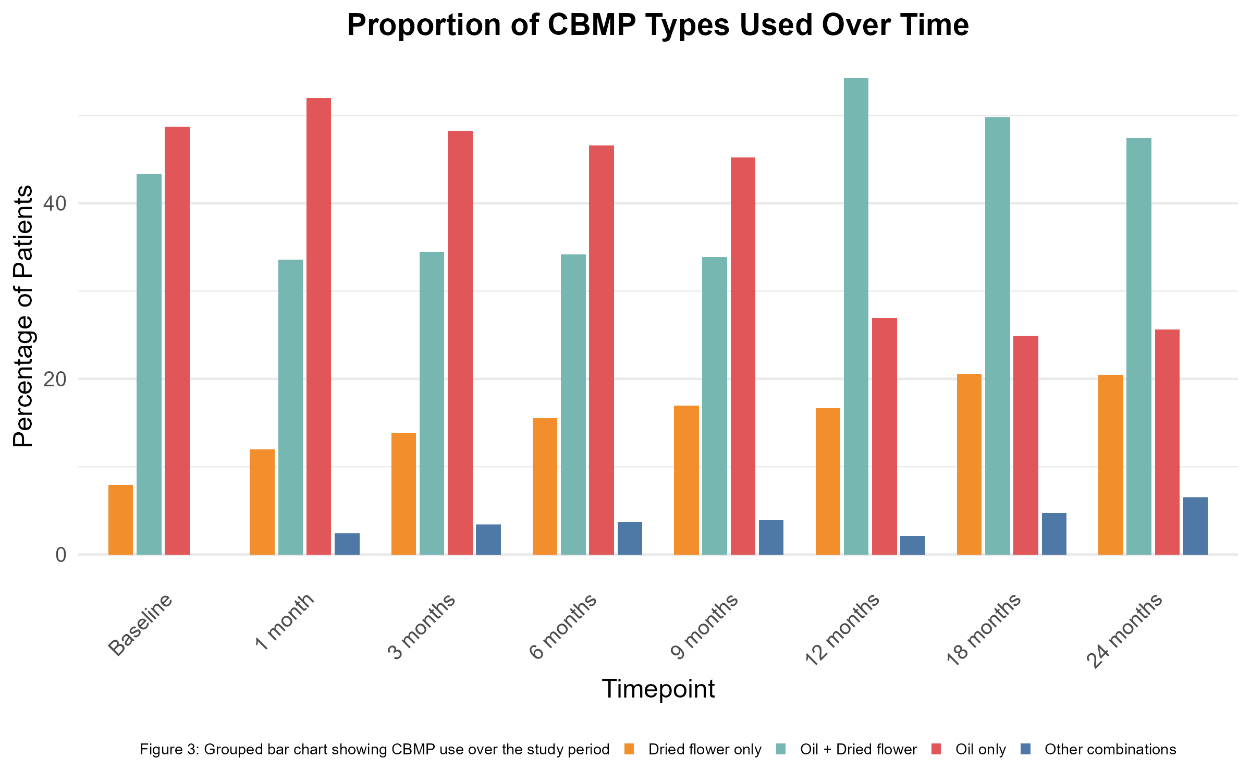
**Supplementary Material**

***Supplementary Figure 1: Proportion of CBMP Types used over time***

*Grouped bar chart showing percentage (%) of participants prescribed different types of cannabis-based medicinal products (CBMPs) at baseline and follow-up intervals.*


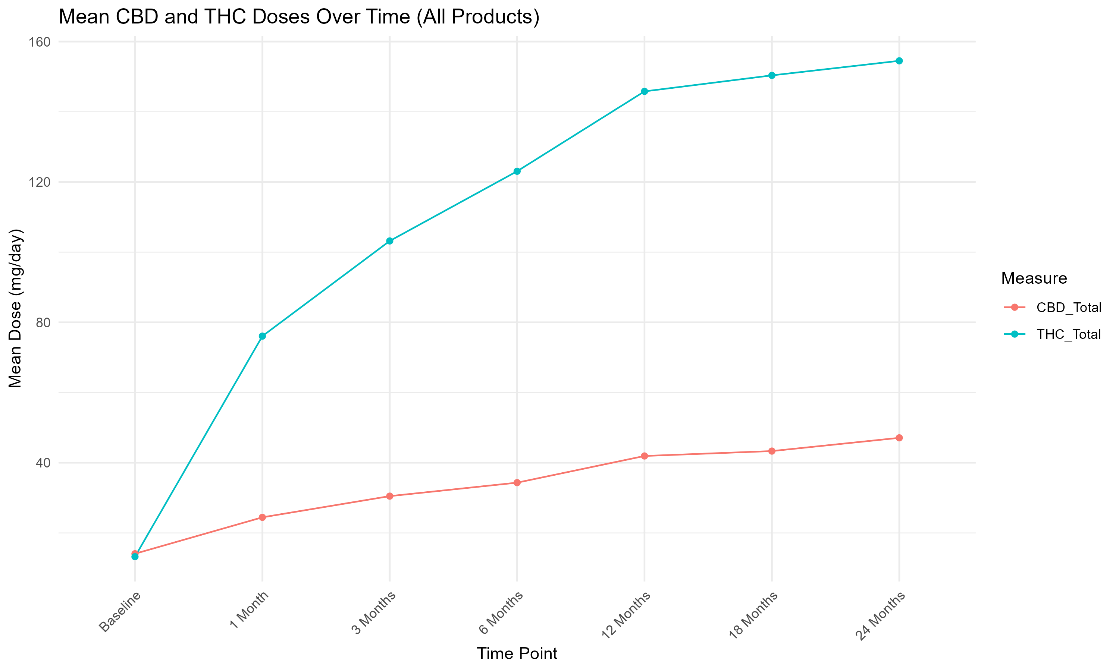


***Supplementary Figure 2: Mean CBD and THC doses over time***

*Graph to show mean doses (mg/day) of (−)-trans-Δ^9^-tetrahydrocannabinol (THC) and cannabidiol (CBD) across 24 months.*


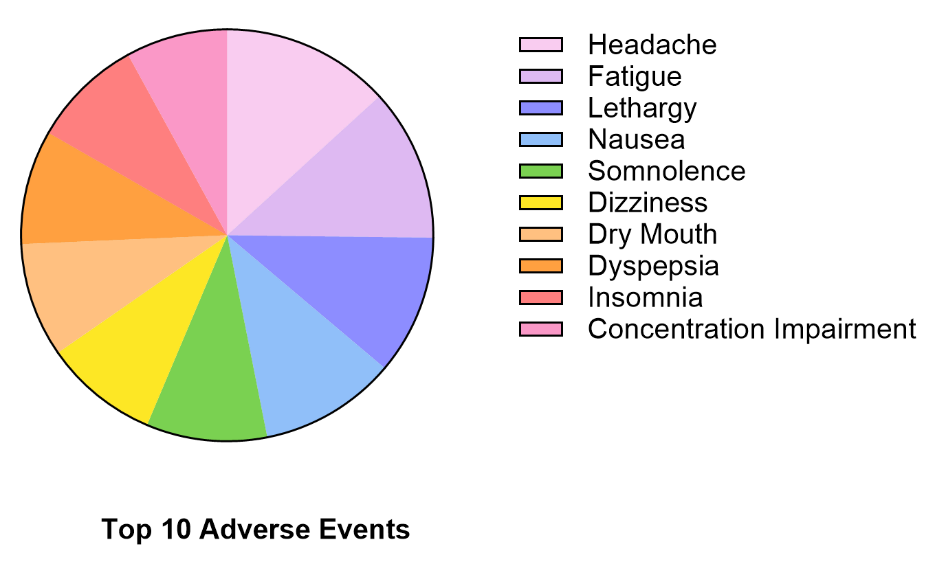


***Supplementary Figure 3: Top 10 Adverse Events over 24 months***

***Supplementary Table 1: Multivariable analysis of Brief Pain Inventory (BPI) Interference scores***

*OR – Odds Ratio, 95% CI – 95% Confidence Interval, BMI – Body Mass Index, CBD – Cannabidiol, THC - (−)-trans-Δ^9^-tetrahydrocannabinol, SQS – Single-item Sleep Quality Scale, GAD-7 – Generalised Anxiety Disorder-7*

| **Predictor** | **OR** | **95% CI** | **p-value** | **Significance** |
| --- | --- | --- | --- | --- |
| Age 31–40 vs <30 | 0.60 | 0.27-1.35 | 0.222 | ns |
| Age 41–50 vs <30 | 1.02 | 0.41-2.56 | 0.958 | ns |
| Age >50 vs <30 | 0.58 | 0.20-1.62 | 0.296 | ns |
| Female vs Male | 1.26 | 0.55-2.84 | 0.578 | ns |
| BMI <20 vs 20–24.99 | 0.28 | 0.11-0.74 | 0.011 | * |
| BMI 25–29.99 vs 20–24.99 | 1.43 | 0.63-3.31 | 0.392 | ns |
| BMI 30–34.99 vs 20–24.99 | 0.40 | 0.15-1.04 | 0.061 | ns |
| BMI ≥35 vs 20–24.99 | 0.66 | 0.25-1.77 | 0.408 | ns |
| Current cannabis user vs Never used | 2.52 | 1.11-5.85 | 0.029 | * |
| Ex-user vs Never used | 1.41 | 0.54-3.76 | 0.488 | ns |
| Dried flower + oil vs Oil | 0.73 | 0.15-3.37 | 0.690 | ns |
| Dried flower vs Oil | 0.86 | 0.16-4.46 | 0.862 | ns |
| Other CBMP vs Oil | 2.14 | 0.43-11.76 | 0.361 | ns |
| CBD Q1–Median vs Min–Q1 | 1.18 | 0.47-3.00 | 0.720 | ns |
| CBD Median–Q3 vs Min–Q1 | 0.72 | 0.29-1.77 | 0.471 | ns |
| CBD Q3–Max vs Min–Q1 | 1.03 | 0.40-2.65 | 0.952 | ns |
| THC Q1–Median vs Min–Q1 | 0.75 | 0.17-3.45 | 0.703 | ns |
| THC Median–Q3 vs Min–Q1 | 0.95 | 0.18-5.13 | 0.954 | ns |
| THC Q3–Max vs Min–Q1 | 0.56 | 0.10-3.06 | 0.501 | ns |
| SQS 0–3 vs 7–10 | 3.94 | 1.46-10.96 | 0.007 | ** |
| SQS 4–6 vs 7–10 | 1.05 | 0.40-2.75 | 0.926 | ns |
| GAD-7 5–9 vs <5 | 1.13 | 0.49-2.62 | 0.769 | ns |
| GAD-7 10–14 vs <5 | 0.75 | 0.29-1.86 | 0.533 | ns |
| GAD-7 ≥15 vs <5 | 0.99 | 0.38-2.58 | 0.990 | ns |

***Supplementary Table 2: Multivariable analysis of Brief Pain Inventory (BPI) Severity scores***

*OR – Odds Ratio, 95% CI – 95% Confidence Interval, BMI – Body Mass Index, CBD – Cannabidiol, THC - (−)-trans-Δ^9^-tetrahydrocannabinol, SQS – Single-item Sleep Quality Scale, GAD-7 – Generalised Anxiety Disorder-7*

| **Predictor** | **OR** | **95% CI** | **p-value** | **Significance** |
| --- | --- | --- | --- | --- |
| Age 31–40 vs <30 | 0.81 | 0.37-1.73 | 0.582 | ns |
| Age 41–50 vs <30 | 0.48 | 0.21-1.11 | 0.088 | ns |
| Age >50 vs <30 | 1.08 | 0.40-3.01 | 0.881 | ns |
| Female vs Male | 1.71 | 0.79-3.74 | 0.174 | ns |
| BMI <20 vs 20–24.99 | 0.34 | 0.13-0.86 | 0.025 | * |
| BMI 25–29.99 vs 20–24.99 | 1.16 | 0.54-2.53 | 0.696 | ns |
| BMI 30–34.99 vs 20–24.99 | 0.58 | 0.22-1.50 | 0.258 | ns |
| BMI ≥35 vs 20–24.99 | 0.42 | 0.16-1.09 | 0.075 | ns |
| Current cannabis user vs Never used | 1.34 | 0.61-2.97 | 0.465 | ns |
| Ex-user vs Never used | 0.86 | 0.34-2.21 | 0.757 | ns |
| Dried flower + oil vs Oil | 0.59 | 0.13-2.54 | 0.483 | ns |
| Dried flower vs Oil | 0.79 | 0.16-3.79 | 0.767 | ns |
| Other CBMP vs Oil | 0.80 | 0.18-3.56 | 0.764 | ns |
| CBD Q1–Median vs Min–Q1 | 1.46 | 0.62-3.47 | 0.389 | ns |
| CBD Median–Q3 vs Min–Q1 | 1.64 | 0.69-3.97 | 0.268 | ns |
| CBD Q3–Max vs Min–Q1 | 1.00 | 0.42-2.41 | 0.998 | ns |
| THC Q1–Median vs Min–Q1 | 0.86 | 0.21-3.81 | 0.841 | ns |
| THC Median–Q3 vs Min–Q1 | 0.66 | 0.14-3.27 | 0.609 | ns |
| THC Q3–Max vs Min–Q1 | 0.59 | 0.12-2.97 | 0.517 | ns |
| SQS 0–3 vs 7–10 | 2.45 | 0.94-6.54 | 0.069 | ns |
| SQS 4–6 vs 7–10 | 1.08 | 0.42-2.83 | 0.876 | ns |
| GAD-7 5–9 vs <5 | 1.19 | 0.53-2.69 | 0.681 | ns |
| GAD-7 10–14 vs <5 | 0.94 | 0.39-2.26 | 0.886 | ns |
| GAD-7 ≥15 vs <5 | 0.97 | 0.39-2.36 | 0.939 | ns |

***Supplementary Table 3: Multivariable analysis of Pain Visual Analogue Scale (VAS) scores***

*OR – Odds Ratio, 95% CI – 95% Confidence Interval, BMI – Body Mass Index, CBD – Cannabidiol, THC - (−)-trans-Δ^9^-tetrahydrocannabinol, SQS – Single-item Sleep Quality Scale, GAD-7 – Generalised Anxiety Disorder-7*

| **Predictor** | **OR** | **95% CI** | **p-value** | **Significance** |
| --- | --- | --- | --- | --- |
| Age 31–40 vs <30 | 0.64 | 0.29-1.41 | 0.271 | ns |
| Age 41–50 vs <30 | 0.64 | 0.41-2.56 | 0.310 | ns |
| Age >50 vs <30 | 0.74 | 0.20-1.62 | 0.574 | ns |
| Female vs Male | 1.00 | 0.55-2.84 | 0.994 | ns |
| BMI <20 vs 20–24.99 | 0.48 | 0.11-0.74 | 0.133 | ns |
| BMI 25–29.99 vs 20–24.99 | 1.57 | 0.63-3.31 | 0.266 | ns |
| BMI 30–34.99 vs 20–24.99 | 0.76 | 0.15-1.04 | 0.570 | ns |
| BMI ≥35 vs 20–24.99 | 0.66 | 0.25-1.77 | 0.386 | ns |
| Current cannabis user vs Never used | 1.16 | 1.11-5.85 | 0.715 | ns |
| Ex-user vs Never used | 1.48 | 0.54-3.76 | 0.439 | ns |
| Dried flower + oil vs Oil | 0.57 | 0.15-3.37 | 0.452 | ns |
| Dried flower vs Oil | 0.69 | 0.16-4.46 | 0.650 | ns |
| Other CBMP vs Oil | 0.99 | 0.43-11.76 | 0.990 | ns |
| CBD Q1–Median vs Min–Q1 | 2.04 | 0.47-3.00 | 0.117 | ns |
| CBD Median–Q3 vs Min–Q1 | 0.84 | 0.29-1.77 | 0.687 | ns |
| CBD Q3–Max vs Min–Q1 | 1.59 | 0.40-2.65 | 0.316 | ns |
| THC Q1–Median vs Min–Q1 | 1.33 | 0.17-3.45 | 0.695 | ns |
| THC Median–Q3 vs Min–Q1 | 1.58 | 0.18-5.13 | 0.576 | ns |
| THC Q3–Max vs Min–Q1 | 0.74 | 0.10-3.06 | 0.707 | ns |
| SQS 0–3 vs 7–10 | 1.86 | 1.46-10.96 | 0.217 | ns |
| SQS 4–6 vs 7–10 | 0.46 | 0.40-2.75 | 0.118 | ns |
| GAD-7 5–9 vs <5 | 1.76 | 0.49-2.62 | 0.184 | ns |
| GAD-7 10–14 vs <5 | 1.24 | 0.29-1.86 | 0.644 | ns |
| GAD-7 ≥15 vs <5 | 0.57 | 0.38-2.58 | 0.216 | ns |

***Supplementary Table 4: Multivariable analysis of Short form - McGill Pain Questionnaire 2 (SF-MPQ-2) scores***

*OR – Odds Ratio, 95% CI – 95% Confidence Interval, BMI – Body Mass Index, CBD – Cannabidiol, THC - (−)-trans-Δ^9^-tetrahydrocannabinol, SQS – Single-item Sleep Quality Scale, GAD-7 – Generalised Anxiety Disorder-7*

| **Predictor** | **OR** | **95% CI** | **p-value** | **Significance** |
| --- | --- | --- | --- | --- |
| Age 31–40 vs <30 | 1.18 | 0.55-2.53 | 0.677 | ns |
| Age 41–50 vs <30 | 2.34 | 0.41-2.56 | 0.053 | ns |
| Age >50 vs <30 | 0.87 | 0.20-1.62 | 0.777 | ns |
| Female vs Male | 1.81 | 0.55-2.84 | 0.127 | ns |
| BMI <20 vs 20–24.99 | 0.46 | 0.11-0.74 | 0.107 | ns |
| BMI 25–29.99 vs 20–24.99 | 1.12 | 0.63-3.31 | 0.765 | ns |
| BMI 30–34.99 vs 20–24.99 | 0.20 | 0.15-0.27 | 0.002 | ** |
| BMI ≥35 vs 20–24.99 | 0.75 | 0.25-1.77 | 0.544 | ns |
| Current cannabis user vs Never used | 0.88 | 1.11-5.85 | 0.752 | ns |
| Ex-user vs Never used | 0.37 | 0.54-3.76 | 0.039 | * |
| Dried flower + oil vs Oil | 0.44 | 0.15-3.37 | 0.299 | ns |
| Dried flower vs Oil | 0.82 | 0.16-4.46 | 0.812 | ns |
| Other CBMP vs Oil | 0.52 | 0.43-11.76 | 0.409 | ns |
| CBD Q1–Median vs Min–Q1 | 0.77 | 0.47-3.00 | 0.553 | ns |
| CBD Median–Q3 vs Min–Q1 | 0.87 | 0.29-1.77 | 0.758 | ns |
| CBD Q3–Max vs Min–Q1 | 0.99 | 0.40-2.65 | 0.982 | ns |
| THC Q1–Median vs Min–Q1 | 1.98 | 0.17-3.45 | 0.373 | ns |
| THC Median–Q3 vs Min–Q1 | 1.26 | 0.18-5.13 | 0.782 | ns |
| THC Q3–Max vs Min–Q1 | 1.67 | 0.10-3.06 | 0.544 | ns |
| SQS 0–3 vs 7–10 | 2.36 | 1.46-10.96 | 0.085 | ns |
| SQS 4–6 vs 7–10 | 0.96 | 0.40-2.75 | 0.939 | ns |
| GAD-7 5–9 vs <5 | 1.37 | 0.49-2.62 | 0.449 | ns |
| GAD-7 10–14 vs <5 | 0.85 | 0.29-1.86 | 0.716 | ns |
| GAD-7 ≥15 vs <5 | 0.84 | 0.38-2.58 | 0.701 | ns |

***Supplementary Table 5: Multivariable analysis Generalised Anxiety Disorder 7 (GAD-7) scores***

*OR – Odds Ratio, 95% CI – 95% Confidence Interval, BMI – Body Mass Index, CBD – Cannabidiol, THC - (−)-trans-Δ^9^-tetrahydrocannabinol, SQS – Single-item Sleep Quality Scale, GAD-7 – Generalised Anxiety Disorder-7*

| **Predictor** | **OR** | **95% CI** | **p-value** | **Significance** |
| --- | --- | --- | --- | --- |
| Age 31–40 vs <30 | 1.54 | 0.60-4.00 | 0.371 | ns |
| Age 41–50 vs <30 | 1.60 | 0.41-2.56 | 0.401 | ns |
| Age >50 vs <30 | 1.55 | 0.20-1.62 | 0.478 | ns |
| Female vs Male | 4.92 | 0.55-2.84 | 0.002 | ** |
| BMI <20 vs 20–24.99 | 1.33 | 0.11-0.74 | 0.626 | ns |
| BMI 25–29.99 vs 20–24.99 | 2.95 | 0.63-3.31 | 0.026 | * |
| BMI 30–34.99 vs 20–24.99 | 0.60 | 0.15-1.04 | 0.424 | ns |
| BMI ≥35 vs 20–24.99 | 4.95 | 0.25-1.77 | 0.010 | * |
| Current cannabis user vs Never used | 2.60 | 1.11-5.85 | 0.065 | ns |
| Ex-user vs Never used | 1.12 | 0.54-3.76 | 0.843 | ns |
| Dried flower + oil vs Oil | 1.97 | 0.15-3.37 | 0.435 | ns |
| Dried flower vs Oil | 2.52 | 0.16-4.46 | 0.340 | ns |
| Other CBMP vs Oil | 10.00 | 0.43-11.76 | 0.016 | * |
| CBD Q1–Median vs Min–Q1 | 4.04 | 0.47-3.00 | 0.014 | * |
| CBD Median–Q3 vs Min–Q1 | 1.68 | 0.29-1.77 | 0.321 | ns |
| CBD Q3–Max vs Min–Q1 | 1.69 | 0.40-2.65 | 0.361 | ns |
| THC Q1–Median vs Min–Q1 | 0.39 | 0.17-3.45 | 0.260 | ns |
| THC Median–Q3 vs Min–Q1 | 0.18 | 0.18-5.13 | 0.080 | ns |
| THC Q3–Max vs Min–Q1 | 0.37 | 0.10-3.06 | 0.307 | ns |
| SQS 0–3 vs 7–10 | 0.89 | 1.46-10.96 | 0.865 | ns |
| SQS 4–6 vs 7–10 | 0.45 | 0.40-2.75 | 0.245 | ns |
| GAD-7 5–9 vs <5 | 9.60 | 0.49-2.62 | <0.001 | *** |
| GAD-7 10–14 vs <5 | 63.58 | 0.29-1.86 | <0.001 | *** |
| GAD-7 ≥15 vs <5 | 71.75 | 0.38-2.58 | <0.001 | *** |

***Supplementary Table 6: Multivariable analysis of Single-item Sleep Quality Scale (SQS) scores***

*OR – Odds Ratio, 95% CI – 95% Confidence Interval, BMI – Body Mass Index, CBD – Cannabidiol, THC - (−)-trans-Δ^9^-tetrahydrocannabinol, SQS – Single-item Sleep Quality Scale, GAD-7 – Generalised Anxiety Disorder-7*

| **Predictor** | **OR** | **95% CI** | **p-value** | **Significance** |
| --- | --- | --- | --- | --- |
| Age 31–40 vs <30 | 1.01 | 0.45-2.27 | 0.989 | ns |
| Age 41–50 vs <30 | 1.15 | 0.41-2.56 | 0.774 | ns |
| Age >50 vs <30 | 1.74 | 0.20-1.62 | 0.320 | ns |
| Female vs Male | 1.20 | 0.55-2.84 | 0.670 | ns |
| BMI <20 vs 20–24.99 | 0.45 | 0.11-0.74 | 0.159 | ns |
| BMI 25–29.99 vs 20–24.99 | 1.00 | 0.63-3.31 | 0.994 | ns |
| BMI 30–34.99 vs 20–24.99 | 0.63 | 0.15-1.04 | 0.367 | ns |
| BMI ≥35 vs 20–24.99 | 0.94 | 0.25-1.77 | 0.900 | ns |
| Current cannabis user vs Never used | 1.54 | 1.11-5.85 | 0.314 | ns |
| Ex-user vs Never used | 1.03 | 0.54-3.76 | 0.953 | ns |
| Dried flower + oil vs Oil | 1.42 | 0.15-3.37 | 0.684 | ns |
| Dried flower vs Oil | 3.26 | 0.16-4.46 | 0.197 | ns |
| Other CBMP vs Oil | 6.37 | 0.43-11.76 | 0.058 | ns |
| CBD Q1–Median vs Min–Q1 | 0.77 | 0.47-3.00 | 0.570 | ns |
| CBD Median–Q3 vs Min–Q1 | 0.81 | 0.29-1.77 | 0.658 | ns |
| CBD Q3–Max vs Min–Q1 | 1.48 | 0.40-2.65 | 0.428 | ns |
| THC Q1–Median vs Min–Q1 | 0.63 | 0.17-3.45 | 0.576 | ns |
| THC Median–Q3 vs Min–Q1 | 0.41 | 0.18-5.13 | 0.341 | ns |
| THC Q3–Max vs Min–Q1 | 0.43 | 0.10-3.06 | 0.361 | ns |
| SQS 0–3 vs 7–10 | >99.99 | 0.00→99.99 | 0.987 | ns |
| SQS 4–6 vs 7–10 | >99.99 | 0.00→99.99 | 0.987 | ns |
| GAD-7 5–9 vs <5 | 1.45 | 0.49-2.62 | 0.432 | ns |
| GAD-7 10–14 vs <5 | 1.96 | 0.29-1.86 | 0.155 | ns |
| GAD-7 ≥15 vs <5 | 2.55 | 0.38-2.58 | 0.052 | ns |

***Supplementary Table 7: Multivariable analysis of EQ-5D-5L Index scores***

*OR – Odds Ratio, 95% CI – 95% Confidence Interval, BMI – Body Mass Index, CBD – Cannabidiol, THC - (−)-trans-Δ^9^-tetrahydrocannabinol, SQS – Single-item Sleep Quality Scale, GAD-7 – Generalised Anxiety Disorder-7*

| **Predictor** | **OR** | **95% CI** | **p-value** | **Significance** |
| --- | --- | --- | --- | --- |
| Age 31–40 vs <30 | 1.23 | 0.56-2.72 | 0.606 | ns |
| Age 41–50 vs <30 | 0.81 | 0.41-2.56 | 0.632 | ns |
| Age >50 vs <30 | 1.28 | 0.20-1.62 | 0.629 | ns |
| Female vs Male | 1.40 | 0.55-2.84 | 0.400 | ns |
| BMI <20 vs 20–24.99 | 1.29 | 0.11-0.74 | 0.593 | ns |
| BMI 25–29.99 vs 20–24.99 | 2.06 | 0.63-3.31 | 0.068 | ns |
| BMI 30–34.99 vs 20–24.99 | 2.48 | 0.15-1.04 | 0.075 | ns |
| BMI ≥35 vs 20–24.99 | 1.94 | 0.25-1.77 | 0.188 | ns |
| Current cannabis user vs Never used | 1.57 | 1.11-5.85 | 0.276 | ns |
| Ex-user vs Never used | 0.83 | 0.54-3.76 | 0.689 | ns |
| Dried flower + oil vs Oil | 2.05 | 0.15-3.37 | 0.349 | ns |
| Dried flower vs Oil | 3.44 | 0.16-4.46 | 0.138 | ns |
| Other CBMP vs Oil | 3.98 | 0.43-11.76 | 0.091 | ns |
| CBD Q1–Median vs Min–Q1 | 1.02 | 0.47-3.00 | 0.966 | ns |
| CBD Median–Q3 vs Min–Q1 | 0.75 | 0.29-1.77 | 0.522 | ns |
| CBD Q3–Max vs Min–Q1 | 0.96 | 0.40-2.65 | 0.940 | ns |
| THC Q1–Median vs Min–Q1 | 0.61 | 0.17-3.45 | 0.499 | ns |
| THC Median–Q3 vs Min–Q1 | 0.76 | 0.18-5.13 | 0.748 | ns |
| THC Q3–Max vs Min–Q1 | 0.69 | 0.10-3.06 | 0.660 | ns |
| SQS 0–3 vs 7–10 | 1.82 | 1.46-10.96 | 0.222 | ns |
| SQS 4–6 vs 7–10 | 1.34 | 0.40-2.75 | 0.549 | ns |
| GAD-7 5–9 vs <5 | 1.35 | 0.49-2.62 | 0.464 | ns |
| GAD-7 10–14 vs <5 | 1.51 | 0.29-1.86 | 0.363 | ns |
| GAD-7 ≥15 vs <5 | 2.13 | 0.38-2.58 | 0.115 | ns |

***Supplementary Table 8: Multivariable analysis of Adverse events***

*OR – Odds Ratio, 95% CI – 95% Confidence Interval, BMI – Body Mass Index, CBD – Cannabidiol, THC - (−)-trans-Δ^9^-tetrahydrocannabinol, SQS – Single-item Sleep Quality Scale, GAD-7 – Generalised Anxiety Disorder-7*

| **Predictor** | **OR** | **95% CI** | **p-value** | **Significance** |
| --- | --- | --- | --- | --- |
| Age 31–40 vs <30 | 1.20 | 0.46-3.24 | 0.708 | ns |
| Age 41–50 vs <30 | 1.69 | 0.61-4.73 | 0.309 | ns |
| Age >50 vs <30 | 3.52 | 1.19-10.93 | 0.025 | * |
| Female vs Male | 1.49 | 0.58-4.25 | 0.423 | ns |
| BMI <20 vs 20–24.99 | 1.13 | 0.37-3.31 | 0.821 | ns |
| BMI 25–29.99 vs 20–24.99 | 1.19 | 0.48-2.93 | 0.702 | ns |
| BMI 30–34.99 vs 20–24.99 | 1.13 | 0.35-3.40 | 0.833 | ns |
| BMI ≥35 vs 20–24.99 | 0.77 | 0.24-2.33 | 0.649 | ns |
| Current cannabis user vs Never used | 0.52 | 0.21-1.25 | 0.144 | ns |
| Ex-user vs Never used | 0.37 | 0.11-1.10 | 0.085 | ns |
| Dried flower + oil vs Oil | 1.52 | 0.31-8.32 | 0.615 | ns |
| Dried flower vs Oil | 0.93 | 0.16-5.75 | 0.933 | ns |
| Other CBMP vs Oil | 0.99 | 0.19-5.09 | 0.989 | ns |
| CBD Q1–Median vs Min–Q1 | 0.46 | 0.15-1.36 | 0.162 | ns |
| CBD Median–Q3 vs Min–Q1 | 0.91 | 0.31-2.68 | 0.867 | ns |
| CBD Q3–Max vs Min–Q1 | 1.83 | 0.66-5.24 | 0.249 | ns |
| THC Q1–Median vs Min–Q1 | 0.66 | 0.12-3.08 | 0.600 | ns |
| THC Median–Q3 vs Min–Q1 | 0.43 | 0.07-2.44 | 0.342 | ns |
| THC Q3–Max vs Min–Q1 | 0.56 | 0.09-3.24 | 0.519 | ns |
| SQS 0–3 vs 7–10 | 0.69 | 0.23-2.16 | 0.507 | ns |
| SQS 4–6 vs 7–10 | 1.45 | 0.50-4.50 | 0.498 | ns |
| GAD-7 5–9 vs <5 | 0.58 | 0.23-1.45 | 0.250 | ns |
| GAD-7 10–14 vs <5 | 0.45 | 0.15-1.31 | 0.150 | ns |
| GAD-7 ≥15 vs <5 | 1.24 | 0.46-3.35 | 0.668 | ns |
